# Novel monoclonal antibodies against pathogenic *Leptospira*

**DOI:** 10.1186/1753-6561-8-S4-P59

**Published:** 2014-10-01

**Authors:** Bruno Moisés de Matos, Cláudia Pinho Hartleben, Leonardo Garcia Monte, Thais Farias Collares, Biancia Sica Siedler, Francine Alves Sinnott

**Affiliations:** 1 Laboratório de Imunodiagnóstico, Biotecnologia, Centro de Desenvolvimento Tecnológico, Universidade Federal de Pelotas, Capão do Leão, Rio Grande do Sul, 96010280, Brazil

## Background

Leptospirosis is a zoonotic disease caused by pathogenic bacteria belonging to the genus *Leptospira *and more than 1 million cases occur worldwide annually. Several mammals may carry the agent, and rats are the most important source of human infection in urban settings. The *Leptospira *surface proteins have an important role during pathogen infection and some of these allow the differentiation between pathogenic and non-pathogenic species. Among these, the LigA and LigB adhesins are surface localized proteins that interact with the proteins of extracellular matrix and fibrinogen. Therefore, antibodies against these targets are useful tools in immunodiagnostic assays.

## Methods

The goal of this study was to generated monoclonal antibodies (mAbs) against a truncated fragment of approximately 54 kDa, named rLigBrep, that comprise a identical portion of LigA and LigB (domains 2-7). For the mAbs production, two BALB/c mice were inoculated via intraperitoneal with 150 µg of rLigBrep on days 0, 14, 21 and 28. Freund's complete adjuvant was used in the first dose and incomplete in the subsequent ones. Four days before cellular fusion the mouse with the highest titer in indirect ELISA (1:64000) was boosted with 20 µg of protein intravenously. Splenic lymphocytes were fused to murine Sp2/O-Ag14 myeloma cells in the presence of PEG 1450. Fused cells were cultivated in Dulbecco's modified Eagle medium containing 20% fetal calf serum and supplemented with hypoxanthine, aminopterin and thymidine (HAT). Hybridomas growing in HAT medium were screened by indirect ELISA and those positive for rLigBrep were cloned twice by limiting dilution, expanded and stored in liquid nitrogen.

## Results and conclusion

Two hibridomas (ID-Ra and ID-Rg) were obtained and used for ascites production. An indirect ELISA was performed to verify antibodies presence in ascites fluid. The mAbs presented high titres against rLigBrep (ID-Ra 51,200 and ID-Rg 128,000, respectively). In conclusion, the mAbs produced in this study can be useful tools in immunodiagnostic assays for leptospirosis.
